# Establishing a green biodesulfurization process for iron ore concentrates in stirred tank and leaching column bioreactors using *Acidithiobacillus thiooxidans*

**DOI:** 10.3389/fbioe.2023.1324417

**Published:** 2023-12-13

**Authors:** Roberto A. Bobadilla-Fazzini, Ignacio Poblete-Castro

**Affiliations:** Biosystems Engineering Laboratory, Department of Chemical and Bioprocess Engineering, Facultad de Ingeniería, Universidad de Santiago de Chile (USACH), Santiago, Chile

**Keywords:** complex iron ores, pyrrhotite, Acidithiobacillus thiooxidans, stirred tank bioreactor, leaching columns

## Abstract

The presence of sulfur impurities in complex iron ores represents a significant challenge for the iron mining and steel-making industries as their removal often necessitates the use of hazardous chemicals and energy-intensive processes. Here, we examined the microbial and mineralogical composition of both primary and secondary iron concentrates, identifying the presence of *Sulfobacillus* spp. and *Leptospirillum* spp., while sulfur-oxidizing bacteria were absent. We also observed that these concentrates displayed up to 85% exposed pyrrhotite. These observations led us to explore the capacity of *Acidithiobacillus thiooxidans* to remove pyrrhotite-sulfur impurities from iron concentrates. Employing stirred tank bioreactors operating at 30°C and inoculated with 5·10^6^ (*At. thiooxidans* cells mL^-1^), we achieved 45.6% sulfur removal over 16 days. Then, we evaluated packed leaching columns operated at 30°C, where the *At. thiooxidans* enriched system reached 43.5% desulfurization over 60 days. Remarkably, sulfur removal increased to 80% within 21 days under potassium limitation. We then compared the *At. thiooxidans*-mediated desulfurization process, with and without air supply, under potassium limitation, varying the initial biomass concentration in 1-m columns. Aerated systems facilitated approximately 70% sulfur removal across the entire column with minimal iron loss. In contrast, non-aerated leaching columns achieved desulfurization levels of only 6% and 26% in the lower and middle sections of the column, respectively. Collectively, we have developed an efficient, scalable biological sulfur-removal technology for processing complex iron ores, aligning with the burgeoning demand for sustainable practices in the mining industry.

## 1 Introduction

Iron ore mining represents the topmost metal extraction activity worldwide, only comparable to the crude petroleum extractive industry. According to the British Geological Survey, iron extraction decreased by 7% from 2017 to 2021, reaching a total of 3.1 billion metric tons (Idoine et al., 2023). This decline aligns with extensive depletion of rich iron ore mines, high iron demand, and exploitation of lower grade iron ores with complex mineralogical composition (Fernández-González et al., 2017). Today, the main iron extracted minerals are hematite (Fe_2_O_3_), goethite (α-FeOOH), and magnetite (Fe_3_O_4_). Typically, these minerals undergo concentration processes in order to enhance the iron content while minimizing impurities, particularly alkalis, sulfur, and phosphorus.

Steel production currently consumes more than 98% of the mined iron ore. The presence of sulfur impurities in iron ore concentrates, particularly when exceeding 0.05%, can lead to steel brittleness and reduced weldability. Therefore, it is imperative to keep sulfur levels as low as possible (Schrama et al., 2017; Rezvanipour et al., 2018). In this sense, the production of iron concentrate utilizing magnetic concentration of Fe_3_O_4_ may result in high sulfur content, primarily due to contamination by pyrrothite (Fe 1-x)S) in the secondary concentrate. Depending on the ore characteristics, including extraction ore and tailings, iron concentrates with varying levels of magnetic sulfur impurities can be produced. The presence of such impurities can result in penalties in iron concentrate sale contracts, with a tolerated sulfur content limit typically set at 0.1% S (Compañía Minera del Pacífico, personal communication). Given these considerations, different approaches have been developed for sulfur impurity removal, including thermal, mechanical, and chemical procedures. One option involves roasting the sulfur content in iron ore using an oxidant, usually oxygen, and heat in a thermal decomposition process. In this operation, sulfur removal is directly related to temperature with significant desulfurization efficiencies achieved above 1,100 C. However, this approach comes with a high carbon footprint, the release of polluting SOx emissions, and changes in ore composition (Abzalov et al., 2008), necessitating different flue gas desulfurization technologies, which incurs significant costs and generate solid waste and wastewater treatment (Xu et al., 2000). Alternatively, the desulfurization of iron ores through flotation leads to a notable loss of valuable minerals. Efforts to enhance efficiency through collector mixtures (Yu et al., 2016) traduce environmental concerns, such as the use of compounds like xanthate (Elizondo-Álvarez et al., 2021). Moreover, the presence of iron ions in solution significantly influences sulfur removal during iron oxide flotation, requiring precise adjustments in each operation to achieve optimal efficiencies (Nakhaei et al., 2019).

A straightforward alternative to iron ore desulfurization involves acid leaching, where the reduction of sulfur content is directly proportional to H_2_SO_4_ concentration and reaction time, while inversely related to particle size. This approach can achieve up to 88% sulfur removal using 4M H_2_SO_4_, 2-h and particle sizes of 10 microns (Ocheri and Mbah, 2016). Additionally, nitric acid leaching has demonstrated remarkable results, with over 80% sulfur conversion from iron ore at 100°C, residence times exceeding 3-h and particle size of 100 µm or smaller (Rezvani Pour et al., 2016). Unfortunately, these experimental conditions often prove economically impractical for industrial applications. Despite the demonstrated leaching ability of acidophilic bacteria to remove sulfur from various ore minerals (Cismasiu, 2010; Shang et al., 2015), especially pit coals (Cismasiu, 2010; Ghosh et al., 2015; Mishra et al., 2020), this technology remains largely unexplored in the iron and steel-making sector. In this regard, *Acidithiobacillus thiooxidans* emerges as the most suitable biotechnological option since it is widely recognized for its acidophilic, aerobic, and chemolithoautotrophic features, and notably, it efficiently uses reduced inorganic sulfur compounds as electron donors while producing sulfuric acid *in situ* (Dopson and Okibe, 2022). These remarkable capabilities offer potential for developing an eco-friendly process to remove sulfur impurities from iron ore concentrates. *At. thiooxidans* has been industrially utilized for cobalt recovery (Morin and d’Hugues, 2007) and for the biooxidation of refractory gold-bearing ores in the BIONORD^®^ process, using stirred bioreactors (Belyi and Tupikina, 2022). Regarding sulfur-oxidizing capacities, our previous research demonstrated that *At. thiooxidans* was more prevalent than *At. ferrooxidans* in mixed cultures (Bobadilla-Fazzini et al., 2011). Additionally, various studies have shown that *At. thiooxidans* bacteria are the dominant species in acidic environments (Okabe et al., 2007; Jones et al., 2012).

Here, we established a biotechnological procedure to efficiently remove sulfur impurities by inoculating the iron concentrate with *A. thiooxidans*. Different bioreactor modes demonstrated high desulfurization rates from iron concentrates offering a novel clean technology for the iron industry.

## 2 Methods

### 2.1 Strains and culture conditions

The *A. thiooxidans* (Bobadilla-Fazzini et al., 2011) used in this study was obtained by enrichment from ore samples from mining regions in Chile. *Acidithiobacillus thiooxidans* was maintained in batch aerated bubble column reactors at 30 C in basal 9Kmedium (990 mg L^−1^ (NH_4_)_2_SO_4_, 145 mg L^−1^ NaH_2_PO_4_·H_2_O, 52 mg L^−1^ KH_2_PO_4_, 100 mg L−1, MgSO_4_·7H_2_O, and 21 mg L^−1^ CaCl_2_), adjusted to pH 1.6 and containing 10 g L^−1^ of elemental sulfur as sole energy source.

### 2.2 Ore concentrate samples

Four different ore concentrate samples, two primary concentrate samples obtained after two rougher magnetic concentration stages and before the classification for grinding denominated “Iron concentrate sample A” (28.39% iron and 1.096% sulfur) and “Iron concentrate sample B” (40.70% iron and 0.950% sulfur), and two from secondary concentrate coming after grinding, flaking and finisher magnetic stages denominated “Iron concentrate sample C” (62,53% iron and 1.200% sulfur) and “Iron concentrate sample D” (61.70% iron and 0.720% sulfur) from an iron mining process in the Atacama Region of Chile were used. The mineralogical ore composition comprises 40.99, 41.63, 81.67% and 82.78% magnetite, 3.21%, 11.05%, 0.35% and 0.74% pyrite, and 0.58, 0.80, 1.42% and 2.10% pyrrhotite, for samples A, B, C and D, respectively. Remaining minerals correspond to gangue including 19.38%, 16.42%, 7.65% and 3.77% plagioclase, 16.51%, 14.10%, 2.19% and 6.28% quartz, 4.16, 3.55, 0.73 and 1.58 epidote for samples A, B, C and D respectively. All samples were received crushed and sieved with 325 Tyler mesh, which means that all particles were below 0.053 mm. Additionally and for bench-scale assays, a primary iron concentrate composite sample generated by standardized mixture of two primary concentrate samples from the same origin as the previous ones was used as “composite sample” (34.40% iron and 0.780% sulfur), with mineralogical ore composition comprising 72.09% magnetite, 5.56% pyrite and 3.78% pyrrhotite.

### 2.3 Bacterial identification and enumeration

Cell number was determined by chamber counting (Thoma Chamber, depth 0.010 mm), counting four fields per sample in triplicate under microscope (Olympus CX31). Strain proportion was determined by specific qPCR determination as previously described (Bobadilla-Fazzini et al., 2011). Briefly, purified genomic DNA was extracted and analyzed with total bacteria 16S rDNA gene region primers (Forward 5′- GTGCCAGCMGCCGCGGTAA -3′, Reverse 5′-CCG​TCA​ATT​CCT​TTG​AGT​TT-3′), rusticyanin gene *rusB* for *At. ferrooxidans* DSM 16786 (Forward 5′-GGA​CAC​CAC​CTG​GAA​AAC -3′, Reverse 5′- TCC​CTT​GTT​GGT​GTT​GAT​G -3′), 16S rDNA gene for *At. thiooxidans* (Forward 5′- TAA​TAT​CGC​CTG​CTG​TTG​AC -3′, Reverse 5′- TTT​CAC​GAC​AGA​CCT​AAT​G-3′), 16S rDNA gene for *Acidiphilium* spp. (Forward 5′- CAACCACGGTCGGGTCAG A-3′, Reverse 5′- TCTCTGACCCGACCGTGG TT-3′), 16S rDNA gene for *Leptospirillum* spp. (Forward 5′- TGA​GGG​GAC​TGC​CAG​CGA​C-3′, Reverse 5′- CTA​GAC​GGG​TAC​CTT​GTT​AC-3′), 16S rDNA gene for *Sulfobacillus* spp. (Forward 5′- CGA​AGG​CGG​TGC​ACT​GGC​C-3′, Reverse 5′- CAG​TGC​ACC​GCC​TTC​GCC​A-3′) and 16S rDNA gene for *Ferroplasma* spp. (Forward 5′- AGA​GTC​AAC​GTC​ACG​AGC​TTA-3′, Reverse 5′- AAG​CTC​GTC​AGG​TTG​ACT​CT-3′).

### 2.4 Sulfur bioconversion assay in stirred bioreactors

To assess the sulfur bioconversion under controlled conditions, 1-L (0.8 L working volume) mechanically agitated bioreactors (BioFlo 110, New Brunswick), including air stripping (0.5 L min^−1^) and temperature control at 30°C and constant agitation 700 rpm, were carried out with 300 g concentrate L^−1^ of secondary ore concentrate samples (samples C and D) and at initial biomass of *At. thiooxidans* of 5·10^6^ (cells mL^−1^).

### 2.5 Sulfur removal assays in leaching column bioreactors

From each ore sample, sub-samples of approximately 500 g were obtained after strict protocols to minimize sampling errors. Each sample was agglomerated with water and inoculum at a dose of 10^6^ (cells g^−1^) and packed in 6 cm diameter 30 cm high acrylic columns fed at a rate of 5 L (h m^2^) ^−1^ with water adjusted to pH 3.0 and addition of 0.5 g (NH_4_)_2_HPO_4_ L^−1^. Assays were done from 7 up to 60 days with forty columns in total, divided in four groups of ten columns each. The first two groups of ten columns each with samples A and B included potassium addition (0.006 g KH_2_PO_4_ L^−1^) as part of the feeding solution (condition N/P/K), while the second group was modified without any potassium addition on the feed (condition N/P), replicated for both primary ore samples. All columns were incubated at 30 C under non-sterile conditions in closed circuit, and evaporation was compensated with pure water.

### 2.6 Scaling up the desulfurization process using leaching columns

For bench-scale assays, the composite sample was sub-sampled in approximately 22 kg after strict protocols to minimize sampling errors. Each sample was agglomerated with water and inoculum at a dose of 10^5^ or 10^6^ cells g^−1^ and packed in 15 cm diameter glass columns fed at a rate of 5 L (h m^2^)^−1^ with water adjusted to pH 3.0 and addition of 0.5 g (NH_4_)_2_HPO_4_ L^−1^. Columns of 1 m were operated for 60 days, both without and with aeration from the base with compressed air at a rate of 0.05 m^3^ (ton h)^−1^. At the end of each assay, each column was drained and discharged, and the iron ores within the column were separated into three equal sections based on lengths at day 60: upper, middle, and lower sections.

### 2.7 Chemical analysis

Total iron was determined after iron ore sample dissolution in HNO3/HCl with heat and serially diluted in volumetric flasks for analysis against standard curve by Atomic Absorption Spectrometry (Perkin Elmer Analyst 400) and Fe(II) ions by the o-phenanthroline method (Kolthoff and Sandell, 1963). Concentrate sulfur content was determined before and after magnetic concentration by Davis Test Tube (DTT) using a LECO 844 series combustion sulfur analyzer. Briefly, 0.1 g of ore concentrate sample was placed in a ceramic crucible and heated to 1,600°C in a stream of purified O_2_. The liberated SO_2_ was then quantified by titration using a standard KIO_3_ solution, with measurements taken by a calibrated automatic SO_2_ titrator (LECO Corp, 2019).

### 2.8 Mineralogical analysis

Before treatment, samples were deagglomerated, sieved, and weighed to obtain exact mass proportions. Grain mounts were prepared, by grinding and polishing prior to optical microscopy. Reflected light microscopy was performed using a Zeiss Axio Image M1m and Axiophot in order to identify the main features of each sample. To determine the mineralogical composition, a statistical point counting method was applied using an integration plate, with a metric network of 400 points, including the analysis of the degree of liberation of the sulfur minerals present, counting the points of free sulfides, associated with gangue and grains included in non-metallic gangue.

## 3 Results

### 3.1 Characterization of the iron ores’ native microbes and mineralogic composition

We first analyzed the presence of native acidophilic microorganisms in the iron ore samples using direct DNA extraction and quantitative PCR (qPCR), following established protocols (Bobadilla-Fazzini et al., 2011). As anticipated, all samples showed high bacteria counts, with no detection of archaea (Table 1). The analysis unveiled that heterotrophic species of the genus *Sulfobacillus* had low abundance in primary concentrate samples A and B, while autotrophic iron-oxidizing *Leptospirillum* spp. was present only in one secondary concentrate sample. Mesophilic bacterial genera such as *Leptospirillum* are widespread due to their reliance on iron oxidation as an energy source. Notably, the detection of *Sulfobacillus* species is somehow atypical, considering their standard classification as moderate thermophilic and with a mixotrophic lifestyle (Bobadilla-Fazzini et al., 2014). However, *Sulfobacillus* species can thrive within a temperature range of 20°C and 60°C (Tat’yana et al., 2006), with *Sulfobacillus acidophilus*, for instance, utilizing ferrous iron as an energy source through autotrophic growth (Norris et al., 1996). Interestingly, mesophilic sulfur-oxidizing *Acidithiobacillus* bacteria were below the detection limit, suggesting their potential use as agents for sulfur removal through inoculation.

**TABLE 1:** Microbiological characterization of iron concentrate ore samples by qPCR.

Name	Total bacteria (cells g^−1^)	*At. ferrooxidans* (cells g^−1^)	*At. thiooxidans* (cells g^−1^)	*Leptospirillum* spp. (cells g^−1^)	*Acidiphilium* spp. (cells g^−1^)	*Ferroplasma* spp. (cells g^−1^)	*Sulfobacillus* spp. (cells g^−1^)	Archaea (cells g^−1^)
Sample A	8.9E+04	n.d^a^	n.d	n.d	n.d	n.d	8.2 E+04	n.d
Sample B	3.6E+04	n.d	n.d	n.d	n.d	n.d	1.7 E+04	n.d
Sample C	2.60E+05	n.d	n.d	1.30E+03	n.d	n.d	n.d	n.d
Sample D	4.0E+03	n.d	n.d	n.d	n.d	n.d	n.d	n.d

^a^ n.d: below detection limit.

Initial mineralogical composition analysis showed that primary iron concentrates had a higher pyrite content compared to secondary concentrate samples, with the latter exhibiting an enrichment in pyrrhotite abundance. This composition indicates that magnetic concentration is not efficient in removing pyrrhotite, making biological treatment a promising technology for eliminating this sulfur impurity. Detailed optical mineralogical analysis in terms of mineral species liberation degree and associations is summarized in the Supplementary Table S1. Key minerals, namely, magnetite, pyrite, and pyrrhotite are predominantly found in their free form. Moreover, a significant proportion of pyrrhotite is associated with pyrite in primary concentrate samples, while pyrrhotite is linked to gangue in the secondary material. This analysis denotes that the weakly magnetic sulfur fraction present in these iron ore concentrate samples is readily accessible to sulfur-oxidizing acidophilic microorganisms. Further, the association of pyrrhotite with pyrite in primary iron ore concentrates is important, considering the possible catalytic galvanic effect that pyrite may exert on the acceleration of leaching kinetics. This phenomenon is commonly observed in various metal sulfides such as sphalerite (Estrada-De los Santos et al., 2016) and enargite (Ma et al., 2021).

### 3.2 Stirred bioreactor sulfur removal from iron ores

**FIGURE 1:**
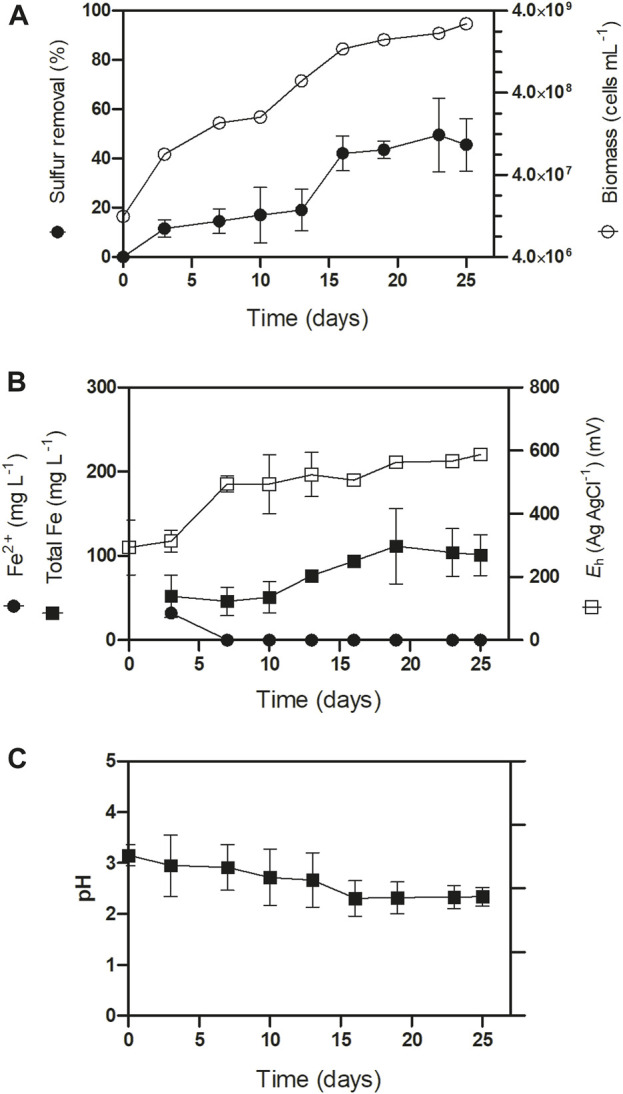
Iron ore secondary concentrate sulfur removal kinetic assays in batch stirred bioreactors with sample “C” and sample “D” at 30°C, 300 (g L^−1^) pulp density with inoculation of At. thiooxidans enriched consortium 5.10^6^ (cells mL^−1^). **(A)** Sulfure removal and biomass, **(B)** Iron content and **(C)** pH of the desulfurization process

Next, we assessed the sulfur conversion ability of *At. thiooxidans* in batch-stirred bioreactors using secondary samples C and D to monitor sulfur conversion over time. The bioreactors started with a 300 (g L^−1^) pulp density and were inoculated to reach a biomass concentration of 5.10^6^ (cells mL^−1^). Due to the sulfur oxidation activity of *At. thiooxidans* and native sulfur-oxidizing bacteria in the ores, the pH gradually decreased over time (Figure 1). The bacterial consortium exhibited a sulfur removal rate of 2.4% ± 0.2% day^−1^ during the first 20 days, reaching a total removal of 45.6% within 16 days (Figure 1A). During this period, we observed minor iron leaching, yielding a low concentration of ∼100 (mg L^−1^). Initially, ferrous iron species were detected in the first 5 days of culturing. Likely attributable to acid-soluble iron species (Figure 1B). Subsequently, the iron was encountered in the ferric form, which persisted until the end of the process (Figure 1B).

### 3.3 Sulfur removal assessment in leaching column bioreactors

The leaching column bioreactor setup closely resembles heap leaching processes. Thus, subsamples of 500 g of primary iron concentrate samples A and B were agglomerated with water and inoculated with *At. thiooxidans* at an initial biomass of 10^6^ (cells g^−1^). Two sets of ten columns each were used for each primary ore sample. A constant feed rate of 5 L (h m^2^)^−1^ was maintained, with the addition of 0.5 g L^−1^ of (NH_4_)_2_HPO_4_ to provide nitrogen and phosphorous (N/P) sources, while avoiding the incorporation of alkali impurities such as sodium and potassium. Based on the nutrient profile previously reported for *At. thiooxidans* (Denisov et al., 1980) a minimum potassium concentration of (6 mg L^−1^ of KH_2_PO_4_) was included (N/P/K) in ten columns for each sample. The assays ran for 60 days totaling 40 columns, 10 columns for each condition: sample A (N/P), sample A (N/P/K), sample B (N/P), and sample B (N/P/K).

**FIGURE 2:**
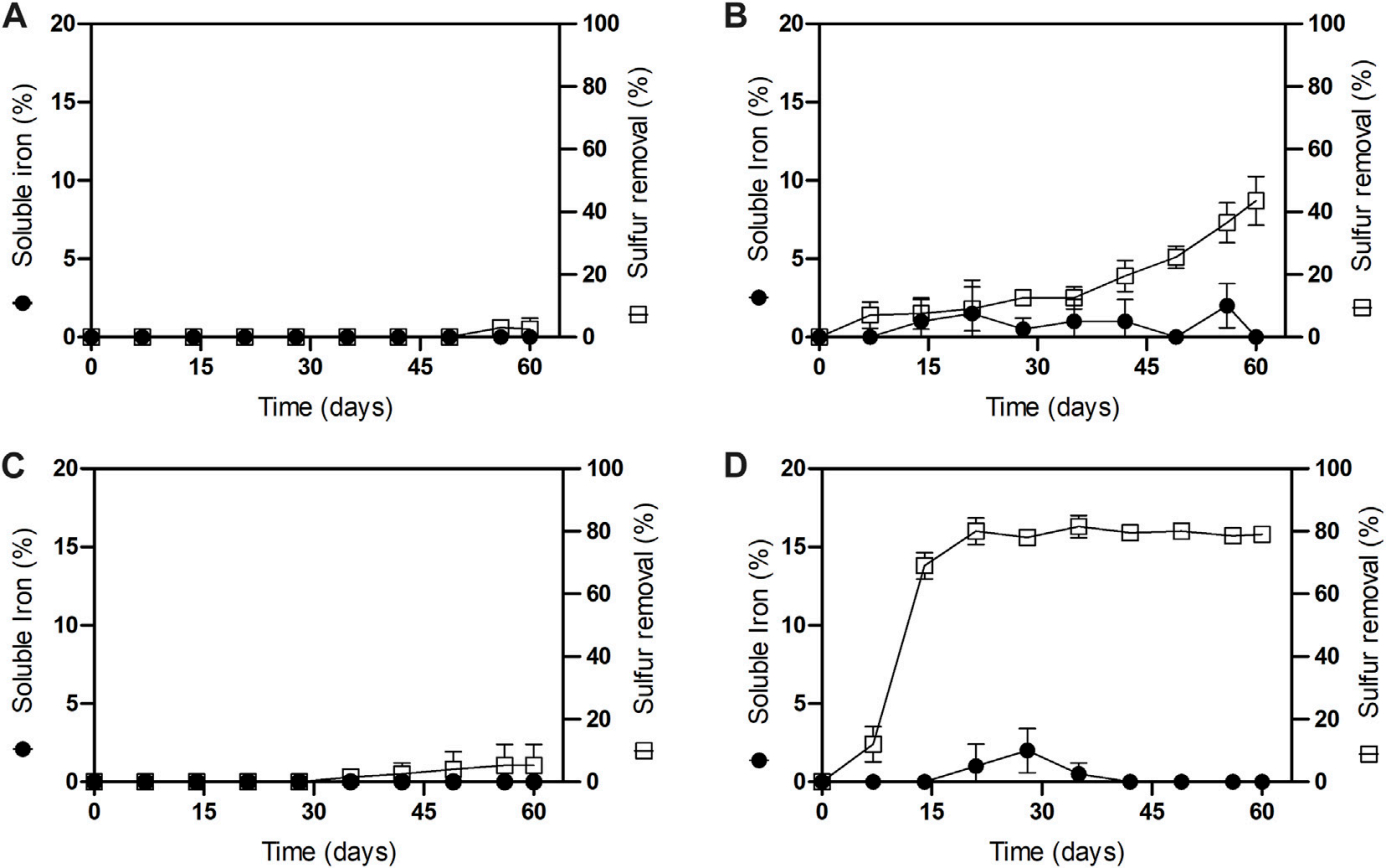
Sulfur removal and iron loss kinetics in iron primary concentrate samples in leaching columns incubated at 30 C inoculated with *At. thiooxidans*. **(A)** N/P/K before magnetic concentration; **(B)** N/P/K after magnetic concentration; **(C)** N/P before magnetic concentration and; **(D)** N/P after magnetic concentration.

To determine sulfur removal kinetics on both iron concentrate samples, with (N/P) and (N/P/K) addition, columns were drained and discharged at 7, 14, 21, 28, 35, 42, 49, 56 and 60 operation days for each condition separately. Dry samples of treated ores were then analyzed for sulfur removal before (Figure 2A; Figure 2C) and after the Davis Test Tube (DTT) for magnetic concentration (Figure 2B; Figure 2D), as well as soluble iron in order to determine iron loss. Figure 2 illustrates the sulfur impurities removal kinetics for (N/P/K) columns, before and after magnetic concentration. The process initially yielded low sulfur removal before magnetic concentration, indicating sulfur enrichment due to precipitation (Figure 2A). After DTT magnetic concentration, the leaching bioprocess resulted in desulfurization yields 43.5% ± 7.8 at day 60, while the loss of iron in solution reached a maximum value of 4% after treatment for this group of columns (Figure 2B).

A series of column assays were conducted without the addition of potassium to prevent the incorporation of alkali impurities. Figure 2C, D also illustrate the kinetics of sulfur impurities removal for columns of the (N/P) group under potassium limitation. In the absence of magnetic concentration, just a small fraction, less than 5%, of sulfur was removed (Figure 2C). Conversely, with DTT concentration, we achieved a sulfur conversion efficiency of 80% ± 4.4 within 21 days as depicted in Figure 2D. Notably, under potassium-depleted conditions, we observed a higher desulfurization activity, effectively doubling the values attained with potassium-replenished conditions. Figure 2D also highlights the loss of iron in (N/P) columns, which reached a maximum of only 3% after treatment, indicating lower values than those found in columns with potassium addition.

### 3.4 Aeration and scalability implications on sulfur removal in bench-scale column bioreactors

**FIGURE 3:**
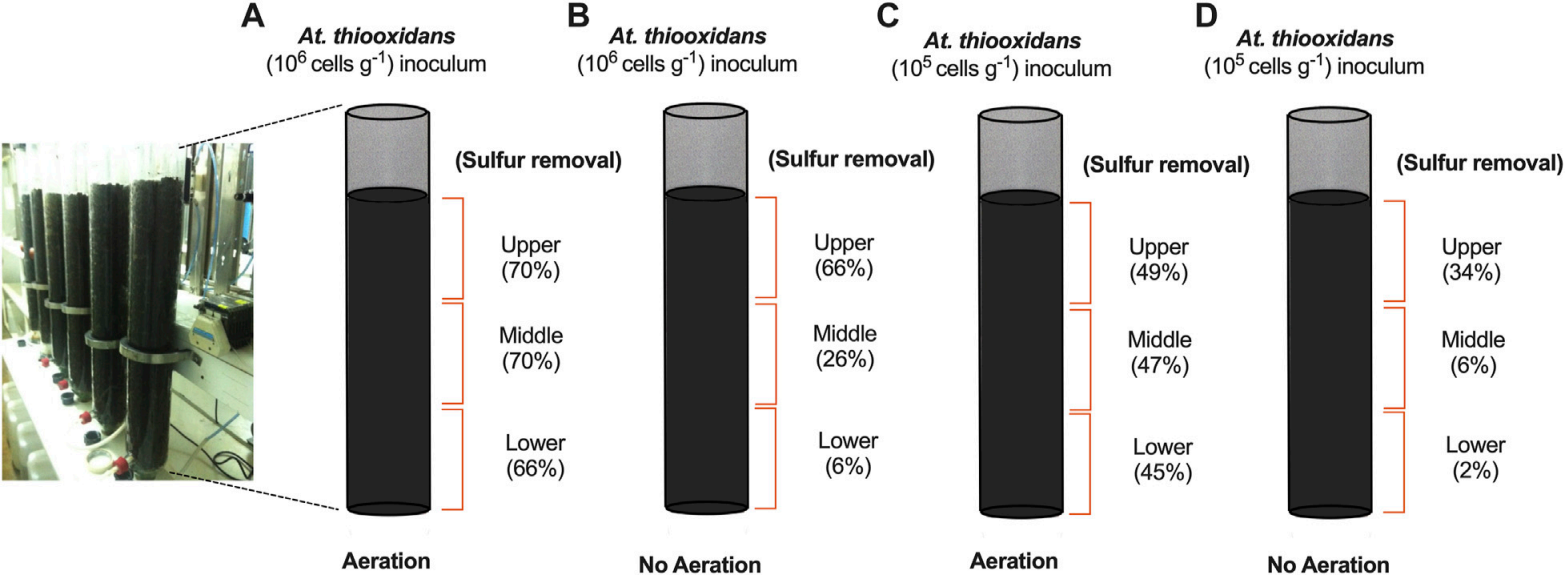
Sulfur removal assay from primary iron ore composite packed column bench-scale bioreactors (1-m length) with (N/P) addition after 60 days operation. **(A)** Aerated column with initial *At. thiooxidans* inoculum 10^6^ (cells g^−1^). **(B)** Non-aerated column with initial *At. thiooxidans* inoculum 10^6^ (cells g^−1^). **(C)** Aerated column with initial *At. thiooxidans* inoculum 10^5^ (cells g^−1^). **(D)** Non-aerated column with initial *At. thiooxidans* inoculum 10^5^ (cells g^−1^).

In our final set of experiments, we extended the length of the column bioreactor to 1 m and monitored sulfur removal in primary iron ore concentrates under potassium limitation. We evaluated two initial cell densities, 10^5^ and 10^6^ (*At. thiooxidans* cells g^−1^), with and without aeration. The supply of oxygen is a critical parameter for aerobic bacterial activity and has been demonstrated to be important in improving leaching efficiency in standard heap leaching operations (Pradhan et al., 2008). Recent findings also demonstrated that aeration can accelerate pyrrhotite oxidation at an industrial scale (Arpalahti and Lundström, 2019). To gain insight into sulfur bioconversion within different sections of the leaching columns, we drained, discharged, and separated the iron ores within the column in three equal lengths at day 60: upper, middle, and lower. Sulfur conversion was quantified for dry sample analysis before and after DTT treatment. The aerated process reached a maximum sulfur removal of nearly 70% across the entire column when *At. thiooxidans* was inoculated at 10^6^ cells g^−1^ (Figure 3A). However, columns without aeration and high initial biomass exhibited low sulfur conversion in the middle and lower part of the column, indicating unfavorable sulfur oxidation activity due to low oxygen availability (Figure 3B). Sulfur removal showed a direct dependence on *At. thiooxidans* inoculation, as aerated columns with lower biomass (10^5^ cells g^−1^) removed only 49% of the available sulfur at day 60 (Figure 3C).

## 4 Discussion

The iron mining industry is currently grappling with major challenges, including soaring demand, the depletion of rich iron ore deposits, and a shift toward the exploitation of complex mineralogical iron ores with escalating sulfur impurities. In this study, we successfully established a desulfurization bioprocess using *At. thiooxidans* in stirred tanks and leaching column bioreactors, achieving 45% and 80% sulfur bioconversion over 20 days of operation, respectively. The pace at which these processes occur is directly correlated with the abundance of microorganisms (Lambert et al., 2014; Tupikina et al., 2014). Based on our results, the higher initial cell density of sulfur-oxidizing bacteria seems to influence the biodesulfurization process in pyrrhotite present in iron concentrates within bench-scale columns. Particularly noteworthy is the observation of enhanced biological sulfur removal under potassium limitation conditions. Inorganic nutrient limitation is known to typically boost the carbon uptake rate and, consequently, the oxidation rate in chemolithoautotrophic (Parro and Moreno-Paz, 2003; Vera et al., 2008), as well as in chemoheterotrophic bacteria (Sulbaran-Bracho et al., 2023).

However, it also exerts a negative impact on biomass formation as this condition can be deleterious for ATP production (Parro and Moreno-Paz, 2003; Vera et al., 2008). Mounting evidence supports the notion that the efficient bacterial sulfur oxidation activities in the leaching process require the development of a complex and orchestrated biofilm matrix. This involves either native or externally introduced acidophiles colonizing the ore surface (Bobadilla-Fazzini, 2014; Dong et al., 2020; Bobadilla-Fazzini and Poblete-Castro, 2021), or relates to the aggregation degree of planktonic cells prior to biofilm formation (Bellenberg et al., 2015; Melaugh et al., 2016; Yavari et al., 2019). Consequently, it is less likely that the improved desulfurization process can be solely attributed to the activity of sulfur-oxidation bacteria. A more plausible explanation lies in the fact that the soluble bioavailable sulfur, existing in intermediate forms between sulfur ion (S^2−^) and sulfate (SO_4_^2−^), such as acid-stable tetrathionate (S_4_O_6_^2−^), partially precipitates in the presence of potassium ions, thereby hindering more efficient sulfur-oxidizing activity. Thus, the exclusion of potassium ions from the leaching solution appears to be a beneficial strategy for achieving increased sulfur removal yields.

The observed kinetics of magnetic sulfur impurity removal from iron concentrate samples during the inoculation of an enriched culture of *At. thiooxidans* reveal transformation reactions driven by oxidation catalysis and precipitation phenomena under the given operating conditions. First, the inoculated sulfur-oxidizing microorganisms, operating under potassium limitation, efficiently catalyze the pyrrhotite (here represented as FeS), a magnetic sulfur impurities oxidation:

$$\text{FeS}+2O_{2}\;\overset{At.\;thiooxidans}{\to }\text{ FeS}O_{4}$$ (1)

This process effectively preserves the non-magnetic sulfur compounds such as pyrite (FeS_2_), while selectively reducing the presence of magnetic sulfur impurities on the iron concentrate. Concurrently, reactions leading to sulfate and/or jarosite precipitation occur, as follows:

$$\begin{array}{l} {\text{FeSO}}_{4}+0.5H_{2}{\text{SO}}_{4}+0.25O_{2}\;\to \;0.5{\text{Fe}}_{2}{\left({\text{SO}}_{4}\right)}_{3}+0.5H_{2}O\\ 3{\text{Fe}}_{2}{\left({\text{SO}}_{4}\right)}_{3}+14H_{2}O\;\to \;2H_{3}{\text{OFe}}_{3}{\left({\text{SO}}_{4}\right)}_{2}{\left(\text{OH}\right)}_{6}+5H_{2}{\text{SO}}_{4}\\ 3{\text{Fe}}_{2}{\left({\text{SO}}_{4}\right)}_{3}+{\text{Na}}_{2}{\text{SO}}_{4}+12H_{2}O\;\to \;2{\text{NaFe}}_{3}{\left({\text{SO}}_{4}\right)}_{2}{\left(\text{OH}\right)}_{6}+6H_{2}{\text{SO}}_{4}\\ 3{\text{Fe}}_{2}{\left({\text{SO}}_{4}\right)}_{3}+K_{2}{\text{SO}}_{4}+12H_{2}O\;\to \;2{\text{KFe}}_{3}{\left({\text{SO}}_{4}\right)}_{2}{\left(\text{OH}\right)}_{6}+6H_{2}{\text{SO}}_{4} \end{array}$$

These precipitation reactions are pH-dependent and result in the form of precipitates on the surface of the iron concentrate. These precipitates must be mechanically removed via a magnetic concentration process, as exemplified by the Davis Test Tube.

When scaling up the bioleaching process in iron ore-packed columns, we found that cell density and air supply are critical process parameters for attaining high sulfur removal across the entire column with minimal iron loss (Figure 3). The feed solution is initially oxygen-saturated, but oxygen rapidly depletes from the draining solution due to aerobic microbial consumption, making it unavailable further downstream in the process (Lizama, 2001). Additionally, there is a linear correlation between increasing temperature and lower oxygen solubility during bioleaching (Huang et al., 2022) along with air convection and diffusion also decreasing along the length of the column, adversely affecting the oxygen-dependent microbial activity essential for bioleaching and the reaction of oxygen with other iron compounds (Casas et al., 1998; Chen et al., 2022). This phenomenon became particularly evident in columns without forced aeration, where we recorded almost negligible sulfur removal at the bottom of the column (Figure 3).

In conclusion, bioleaching columns that are supplied with compressed air and inoculated with an initial cell concentration of *At. thiooxidans* at 10^6^ cells g^−1^ under potassium limitation present the most suitable condition for converting pyrrhotite-sulfur impurities in iron concentrates. This bioprocess emerges as a sustainable alternative to conventional methods that typically involve the use of contaminating chemicals and energy-consuming procedures.

## Data Availability

The raw data supporting the conclusion of this article will be made available by the authors, without undue reservation.
