# Supplementary material for: Establishing a green biodesulfurization process for iron ore concentrates in stirred tank and leaching column bioreactors using Acidithiobacillus thiooxidans
Source: Front Bioeng Biotechnol. 2023 Dec 13;11:1324417. doi: 10.3389/fbioe.2023.1324417 (PMC10751661; doi:10.3389/fbioe.2023.1324417)
Supplement: Supplementary file 1 [file Table1.DOCX]

**Supplementary material**

Table 1S. Liberation analysis of iron ore concentrate samples

| **Name** | **Minerals** | **Free**  **%** | **Assoc to Mt**  **%** | **Incl in Gg**  **%** | **Assoc to Hm**  **%** | **Assoc to Cp**  **%** | **Assoc to Gg**  **%** | **Assoc to Py**  **%** | **Assoc to Po**  **%** | **Mixed**  **%** | **Gg Incl in**  **%** |
| --- | --- | --- | --- | --- | --- | --- | --- | --- | --- | --- | --- |
| **Sample A** | **Mt** | 83.35 | -- | 0.61 | 0.95 | 0.61 | 11.39 | 0.91 | 0.57 | 1.22 | 0.38 |
|  | **Py** | 82.35 | 9.80 | -- | -- | 1.96 | -- | -- | 5.88 | -- | -- |
|  | **Po** | 60.00 | 10.00 | -- | -- | -- | 10.00 | 20.00 | -- | -- | -- |
| **Sample B** | **Mt** | 81.92 | -- | 2.05 | 0.35 | 0.34 | 10.82 | 0.34 | -- | 3.99 | 0.18 |
|  | **Py** | 92.12 | 7.27 | -- | -- | -- | -- | -- | 0.61 | -- | -- |
|  | **Po** | 84.62 | -- | -- | -- | -- | -- | 15.38 | -- | -- | -- |
| **Sample C** | **Mt** | 96.55 | -- | 0.93 | -- | -- | 2.01 | 0.15 | -- | 0.26 | 0.04 |
|  | **Py** | 78.26 | 4.35 | -- | -- | -- | -- | -- | 17.39 | -- | -- |
|  | **Po** | 74.79 | 3.36 | -- | -- | -- | 9.24 | 3.36 | -- | -- | -- |
| **Sample D** | **Mt** | 99.14 | -- | 0.23 | -- | -- | 0.50 | 0.04 | -- | 0.06 | 0.01 |
|  | **Py** | 75.00 | 5.00 | -- | -- | -- | -- | -- | 20.00 | -- | -- |
|  | **Po** | 82.41 | 3.70 | -- | -- | -- | 10.19 | 3.70 | -- | -- | -- |

Abbreviations: Po: pyrrhotite; Py: pyrite; Mt: magnetite; Hm: hematite; Cp: chalcopyrite; Gg: gangue. Assoc: associated; Incl: included.
